# Acupuncture as a treatment for functional dyspepsia: design and methods of a randomized controlled trial

**DOI:** 10.1186/1745-6215-10-75

**Published:** 2009-08-23

**Authors:** Hui Zheng, Xiao-ping Tian, Ying Li, Fan-rong Liang, Shu-guang Yu, Xu-guang Liu, Yong Tang, Xu-guang Yang, Jie Yan, Guo-jie Sun, Xiao-rong Chang, Hong-xing Zhang, Ting-ting Ma, Shu-yuan Yu

**Affiliations:** 1 Chengdu University of Traditional Chinese Medicine, Chengdu, Sichuan, PR China; 2 Hunan University of Traditional Chinese Medicine, Changsha, Hunan, PR China; 3 Hubei College of Traditional Chinese Medicine, Wuhan, Hubei, PR China; 4 No.1 People's Hospital of Wuhan City, Wuhan, Hubei, PR China

## Abstract

**Background:**

Acupuncture is widely used in China to treat functional dyspepsia (FD). However, its effectiveness in the treatment of FD, and whether FD-specific acupoints exist, are controversial. So this study aims to determine if acupuncture is an effective treatment for FD and if acupoint specificity exists according to traditional acupuncture meridians and acupoint theories.

**Design:**

This multicenter randomized controlled trial will include four acupoint treatment groups, one non-acupoint control group and one drug (positive control) group. The four acupoint treatment groups will focus on: (1) specific acupoints of the stomach meridian; (2) non-specific acupoints of the stomach meridian; (3) specific acupoints of alarm and transport points; and (4) acupoints of the gallbladder meridian. These four groups of acupoints are thought to differ in terms of clinical efficacy, according to traditional acupuncture meridians and acupoint theories. A total of 120 FD patients will be included in each group. Each patient will receive 20 sessions of acupuncture treatment over 4 weeks. The trial will be conducted in eight hospitals located in three centers of China. The primary outcomes in this trial will include differences in Nepean Dyspepsia Index scores and differences in the Symptom Index of Dyspepsia before randomization, 2 weeks and 4 weeks after randomization, and 1 month and 3 months after completing treatment.

**Discussion:**

The important features of this trial include the randomization procedures (controlled by a central randomization system), a standardized protocol of acupuncture manipulation, and the fact that this is the first multicenter randomized trial of FD and acupuncture to be performed in China. The results of this trial will determine whether acupuncture is an effective treatment for FD and whether using different acupoints or different meridians leads to differences in clinical efficacy.

**Trial registration number:**

Clinical Trials.gov Identifier: NCT00599677.

## Background

Functional dyspepsia (FD) is defined as chronic upper abdominal discomfort or pain, heartburn, early satiety, nausea, vomiting, abdominal distension and bloating, without any apparent organic, systemic, or metabolic pathology to explain these symptoms [1-3]. An epidemiological survey of Western countries showed that the prevalence of FD ranges from 11.5% to 14.7% [4]. An additional study conducted in Tianjin, China, revealed that 23.29% of the population suffers from FD [5]. A common and well-recognized public health problem, FD reduces patient quality of life and increases the economic burden of medical care [6-11].

Although several classes of drugs (such as proton pump inhibitors, H2-blockers, and prokinetic drugs) have been used in clinical practice and some (such as antisecretory agents or prokinetics) have been effective in placebo-controlled trials, only a small proportion of patients report sufficient relief from these treatments [12].

The exact etiology of FD remains unclear; thus, it is not surprising that current treatments for FD are of limited efficacy and that alternative therapies, including acupuncture, are attractive to both patients and practitioners [13]. Acupuncture has been used in China for many years to treat gastrointestinal symptoms, including acute and chronic gastroenteritis, diarrhea, constipation and gastroduodenal ulcers [14]. Several randomized controlled trials (RCTs) have suggested that acupuncture is an effective treatment for FD, particularly with regards to dyspeptic symptoms, belching and anorexia [15-17]. However, it has been difficult to draw firm conclusions from the results of these RCTS due to the poor quality of the studies and the small sample sizes. Therefore, the mechanisms by which acupuncture treat FD, or whether this therapy works at all, remain the subject of debate. Therefore, we have designed a multicenter randomized controlled trial to address these problems and provide more conclusive data on the use of acupuncture to treat FD.

The first aim of this trial will focus on the efficacy of acupuncture as a treatment for FD. Second, we will attempt to determine if the efficacy of acupuncture differs with the type of acupoint, or with the use of acupoints for different meridians. In a separate paper, we have described the methods of a clinical trial to examine the role of acupuncture in managing migraines [18], and we are currently conducting a similar study to assess the efficacy of acupuncture as a treatment for migraines.

## Methods

### Study design

**Figure 1:**
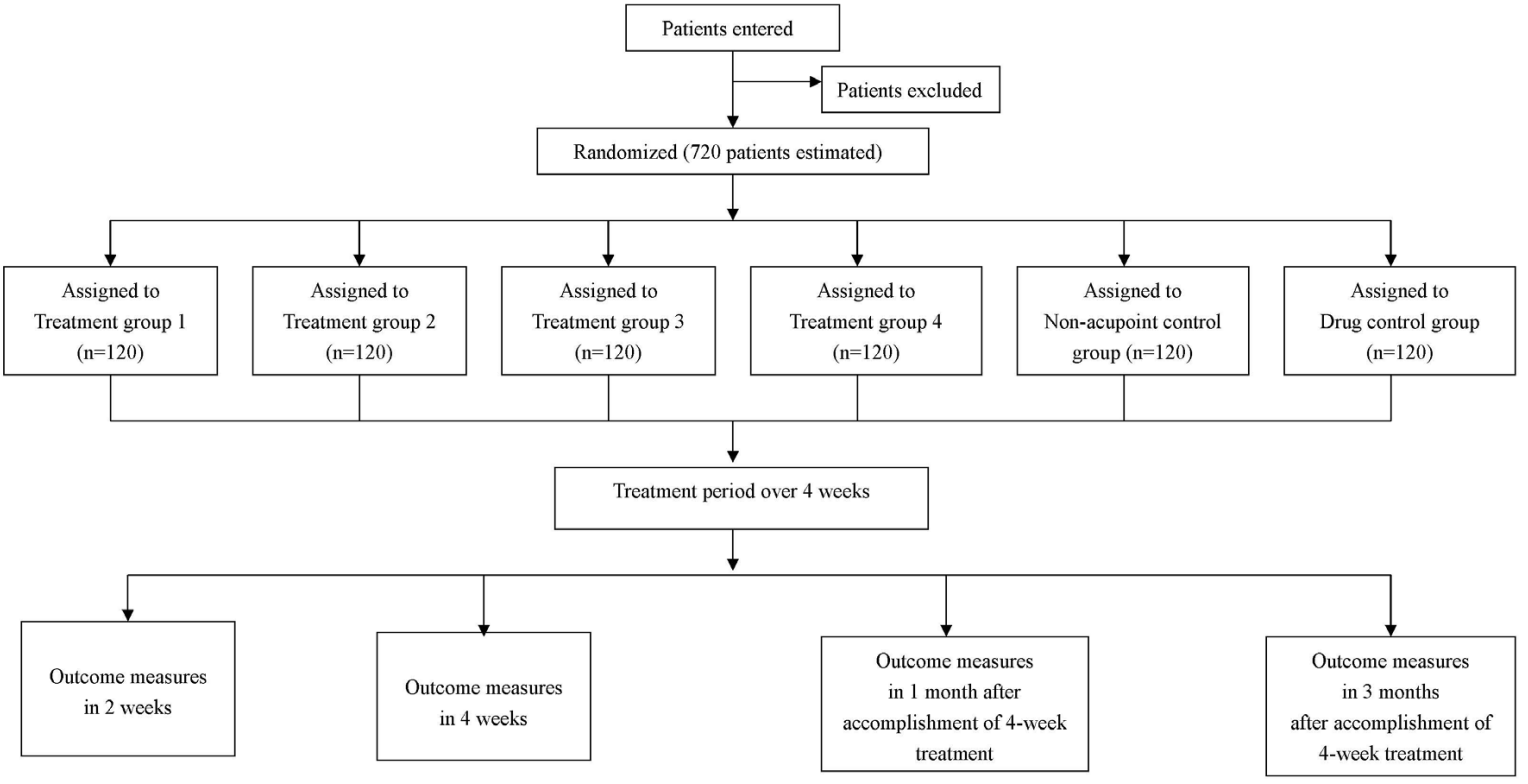
**Trial flow chart**.

This study will consist of a multicenter randomized controlled trial to compare four acupoint treatment groups with one non-acupoint control group and one drug (positive control) group (Figure 1, Table 1). This trial will be completed in eight hospitals, including the First Affiliated Hospital of Chengdu University of Traditional Chinese Medicine (TCM), People's Hospital of Sichuan Province, No. 4 People's Hospital of Sichuan Province, Affiliated Hospital of Ningxia Medicine University, First Affiliated Hospital of Hunan University of TCM, No. 1 People's Hospital of Wuhan City, TCM Hospital of Hubei Province, and the TCM Hospital of Wuhan City.

**Table 1:** Time to visit and data collection

	-1 week	0 week	2 week	4 week	1 month after Treatment Phase	3 months after Treatment Phase
			
	Baseline	Treatment phase			Follow-up Phase	
**Patients**						
Informed consent	X					
Medical history	X					
Physical examination	X					
Randomization		X				
						
**Interventions**						
Specific acupoints of Stomach meridians(n = 120)						
Non-specific acupoints of Stomach meridians(n = 120)		20 sessions of acupuncture at acupoints				
Acupoints of Shu and Mu acupoints(n = 120)						
Acupoints of Gallbladder meridians(n = 120)						
						
**Comparisons**						
Non-acupoints control group(n = 120)		20 sessions of acupuncture at non-acupoints				
Itopride control group(n = 120)		50 mg three times daily, 20 days treatment				
						
**Outcomes**^a^						
NDI		X	X	X	X	X
SID		X	X	X	X	X
SF-36		X		X	X	X
						
**Participants safety**						
Laboratory test		X		X		
Adverse events		X	X	X	X	X

a. NDI = Nepean Dyspepsia Index; SID = Symptom Index of Dyspepsia; SF-36 = Mos 36-item Short Form Health Survey

This trial will include a 4-week treatment period and a 3-month follow-up period. After randomization, patients will receive 20 treatments over a period of 4 weeks. Outcome measurements will be assessed at baseline (i.e., 1 week after subjects are diagnosed with FD), as well as 2 weeks and 4 weeks after randomization, and 1 month and 3 months after conclusion of the treatment phase. Patients will be informed that they may be assigned to an acupuncture group, a non-acupoint control group, or the itopride control group.

This trial will be performed according to the principles of the Declaration of Helsinki (Version Edinburgh 2000). The study protocol has been approved by regional ethics review boards, including the national review board for clinical drug research, located in the Chengdu University Hospital of Traditional Chinese Medicine. This study protocol has permission number 2007 KL-002. All patients will be asked to provide written consent before enrollment, and will be given ample time to decide if they wish to participate and sign the consent form. For subjects who do not want to participate in acupuncture, other treatment options will be available.

### Randomization

A central-randomization scheme will be performed at the Chengdu Good Clinical Practice (GCP) Center, which is affiliated with the National Clinical Trial Center of Chinese Medicine. This scheme will be used to assign patients to one of six groups in a 1:1:1:1:1:1 ratio. A permuted-block randomization procedure, which is automatically under the control of a central computer system, will be used to generate an allocation sequence and blind researchers to the group assignments. Patients who meet the inclusion criteria will be referred to acupuncturists at one of the eight study hospitals. The cell phones of all acupuncturists will be approved and assigned specific ID numbers after the acupuncturist completes a special training course designed for this trial. The acupuncturists will then use the registered cell phones to send randomization information to the GCP Center. This randomization information will include the subject's name in a pinyin format, the subject's numerical birthday and the subject's gender. Once the GCP Center computer system receives the randomization information, it will randomly assign patients to an acupoint treatment group or to the control group. Random numbers and group assignments will be sent from the GCP Center to the acupuncturists via e-mail or short message service (SMS). This procedure will ensure adequate randomization concealment. Patients will be blinded to the type of acupuncture treatment they receive.

### Blinding

As the acupuncturists are responsible for sending randomization information and receiving notices of group assignments, it would not be practical to blind the acupuncturists to treatment allocations and patients' symptoms. Additionally, it is not possible to prevent patients from knowing if they have received acupuncture or itopride treatment. However, it is feasible and ethical to blind patients to the type of acupuncture they receive. In addition, we intend to conceal the group assignments from outcome assessors and our statistician. To assist with the blinding procedure, we named the groups A, B, C, D, E, and F in the medical records and case report forms (CRFs). The specific acupoints (or non-acupoints) will not be mentioned in these forms.

### Eligibility

#### Inclusion criteria

According to the Rome III Diagnostic Criteria for Functional Gastrointestinal Disorders [19], FD can be diagnosed by: (1) meal-induced dyspeptic symptoms (PDS); and (2) epigastric pain (EPS). Patients diagnosed with PDS or EPS will be considered for inclusion in this trial. Patients with one or more of these symptoms (such as postprandial fullness, early satiation, or epigastric pain or burning) will be considered patients with dyspepsia. Accordingly, the inclusion criteria for this study are consistent with the diagnostic criteria for functional dyspepsia: aged between 18 and 65 years; not received gastroenteric dynamic drugs in the 15 days before enrollment; not be taking part in any other clinical trials; and provide written informed consent.

#### Exclusion criteria

Patients with any of the following conditions will be excluded: contraindications to itopride, unconsciousness, history of mental disorders, aggravating malignant tumors or other serious consumptive disease, susceptibility to infection and bleeding, serious diseases of the cardiovascular, hepatic, renal, gastrointestinal, hematological systems, pregnancy or lactation.

### Recruitment strategies

#### Hospital-based recruiting

A large number of patients with FD are currently receiving healthcare or seeking health advice at hospitals in China. The host institution developed collaborations with eight additional hospitals. Primary care physicians at these hospitals are familiar with the study inclusion and exclusion criteria. Interested patients will be referred to specific researchers at a study hospital, who will obtain informed consent and conduct the screening evaluations. Patients who are willing to participate and who satisfy the inclusion criteria will be introduced to acupuncturists at the eight hospitals.

#### Television and newspaper advertisements

Television advertisements will be broadcast on local channels to promote study participation. Advertisements will also be published in local newspapers. Printed recruitment posters will be posted in the eight participating hospitals.

### Interventions

Interventions were designed according to records in ancient books and according to recent studies of acupuncture as a treatment for FD. Itopride is a non-acupuncture treatment option in this trial. The acupuncture protocol was developed in consensus with Chinese acupuncturists and acupuncture experts, and is consistent with the STRICTA (standards for reporting interventions in controlled trials of acupuncture) guidelines for the performance of acupuncture studies [20].

### Rationale for selecting acupoints

The acupuncture treatment protocol is consistent with the principles of traditional acupuncture theories. These principles state that FD is in close proximity to the stomach meridian; hence, it is reasonable that the acupoint corresponding to the stomach meridian would be most effective at treating this condition. We also chose to study the gallbladder meridian because it is anatomically close to the stomach meridian and therefore may achieve similar effects. Furthermore, traditional acupuncture theories maintain that specific points convey specific therapeutic effects. Thus, the specific acupoint corresponding to the stomach meridian would be expected to be more effective than a non-specific acupoint of the same meridian. Therefore, we selected the most frequently used specific and non-specific acupoints of the stomach meridian, according to the results of a previous review of ancient and modern literature. According to traditional acupuncture theories, the alarm and transport points are considered specific points. Alarm points (Front-Mu points) are specific points on the chest or abdomen where the qi of the respective viscera are concentrated, whereas transport points (Back-Shu points) are specific points on the back where the qi of the visceral organs are infused. Both alarm points and transport points can be used to treat diseases of specific Zang-Fu organs; for example, Zhongwan (CV12) and Weishu (BL21) are the alarm and transport points of the stomach and can be used to treat diseases related to this organ. Thus, Zhongwan (CV12) and Weishu (BL21) were selected for use in one of the acupuncture treatment groups.

### Control groups

Because itopride (Shenzhen Tsinghua Yuanxing Pharmaceutical Co., Ltd.) is an effective treatment for FD [21], we chose to use this drug as a positive control. Patients in the positive control group will receive similar treatment sessions as acupuncture groups, with administration of 50 mg itopride, three times daily, taken half an hour before meals [21].

**Figure 2:**
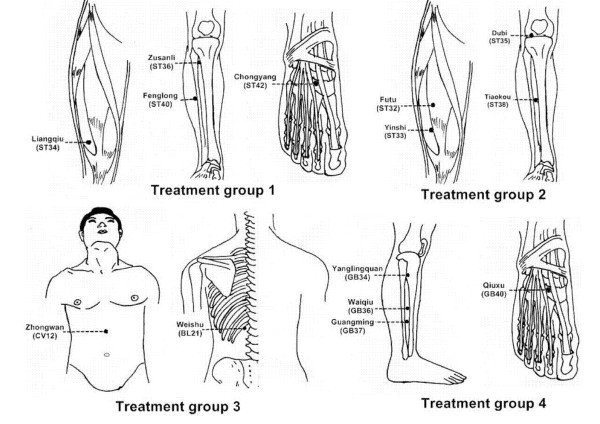
**Locations of acupoints (acupuncture treatment groups)**. Acupoints of treatment group 1 are specific acupoints of Stomach meridian. Acupoints of treatment group 2 are non-specific acupoints of Stomach meridian. Acupoints of treatment group 3 are Alarm and transport points. Acupoints of treatment group 4 are acupoints of Gallbladder meridian.

**Figure 3:**
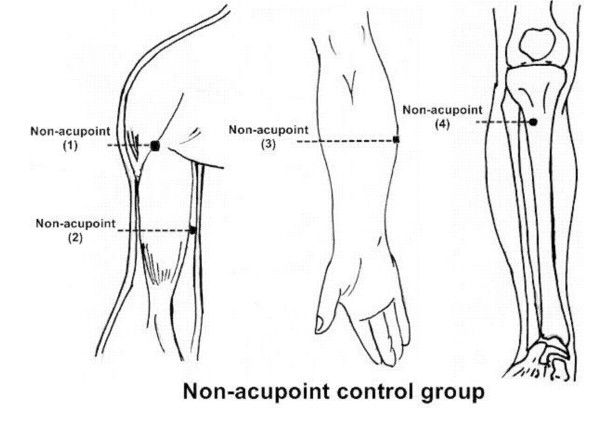
**Locations of non-acupoints (acupuncture control group)**. Descriptions of locations non-acupoints are available at table 2.

The locations of non-acupoints have been defined in the literature [22-24]. The locations and manipulations of acupoints and non-acupoints are shown in Table 2, Figure 2 and Figure 3.

**Table 2:** Details of each group.

Group	Acupoints	Manipulation
**Treatment group 1** (specific acupoints of stomach meridian)	(1) Chongyang(ST42)	(1) is punctured perpendicularly 0.3–0.5 cun avoiding the artery
	(2) Fenglong(ST40)	(2) is punctured perpendicularly 1–1.5 cun
	(3) Zusanli(ST36)	(3) is punctured perpendicularly 1–2 cun
	(4) Liangqiu(ST34)	(4) is punctured perpendicularly 1–1.2 cun
**Treatment group 2** (non-specific acupoints of Stomach meridian)	(1) Tiaokou(ST38)	(1) is punctured perpendicularly 1–1.5 cun
	(2) Dubi(ST35)	(2) is punctured perpendicularly 0.5–1 cun
	(3) Yinshi(ST33)	(3) is punctured perpendicularly 1–1.5 cun
	(4) Futu(ST32)	(4) is punctured perpendicularly 1–2 cun
		
**Treatment group 3** (Alarm and transport point)	(1) Weishu(BL21)	(1) is punctured obliquely along the spine fo r 0.5–0.8 cun
	(2) Zhongwan(CV12)	(2) is punctured perpendicularly 1–1.5 cun
**Treatment group 4** (acupoints of Gallbladder meridian)	(1) Qiuxu(GB40)	is punctured perpendicularly 0.5–0.8 cun
	(2) Guangming(GB37)	(2) is punctured perpendicularly 0.5–0.8 cun
	(3) Yanglingquan(GB34)	(3) is punctured perpendicularly 1–1.5 cun
	(4) Waiqiu(GB36)	(4) is punctured perpendicularly 0.5–0.8 cun
		
**Non-acupoint group**	(1) At the medial arm on the anterior border of the insertion of the deltoid muscle at the junction of deltoid and biceps muscles	(1) is punctured perpendicularly 0.5–1 cun
	(2) Half between the tip of the elbow and the axillae	is punctured perpendicularly 0.5–1 cun
	(3) Ulnar side, half between epicodylus medialis of the humerus and ulnar side of the wrist	(3) is punctured perpendicularly 0.5–1 cun
	(4) The edge of tibia 1 to 2 cm lateral to the Zusanli(ST36) horizontally	(4) is punctured perpendicularly 0.5–1 cun

### Standardized protocol for acupuncture manipulation

Sterile disposable stainless steel needles (length: 25–40 mm, diameter: 0.25 mm; Hwatuo, Suzhou, China) will be used on patients in the acupuncture group. Except for the CV12 acupoint, all acupoints and non-acupoints will be punctured unilaterally, alternating between the left and the right to relieve the pain caused from the insertion of two needles into one acupoint. All acupoint and non-acupoint control groups will receive electro-stimulation of acupuncture points. The technique used in the non-acupoint control group will be the same as treatment groups, except that no attempt will be made to achieve the *Deqi *sensation. After the stimulation of each acupoint or non-acupoint, auxiliary needles (13 mm in length and 0.18 mm in diameter) will be inserted laterally (proximal limbs or trunk) into acupoints or non-acupoints to a depth of 2 mm without manual stimulation. Therefore, eight needles will be inserted into each patient. Transcutaneous electric acupoint stimulation (Han's acupoint nerve stimulator, HANS, Model LH 200A TENS, Nanjing, China) will be used for the electro-acupuncture stimulation of every acupoint or non-acupoint after needle insertion. Each acupuncture needle and its auxiliary needle will be connected to the HANS electrical leads for 30 minutes, with a stimulation frequency of 2/100 Hz. The stimulation intensity will vary from 0.1 mA to 1.0 mA for all patients. Electro-acupuncture will be used to ensure that patients in different acupuncture groups receive the same extent of stimulation, which will ensure consistency with regards to performance. Other interventions (such as moxibustion, cupping and herbs) will not be used.

Patients in the acupuncture treatment groups and the non-acupoint control group will receive a total of 20 treatments over a 4-week period. The treatments will be administered once per day, for 5 continuous days, with a 2 day rest interval. This treatment course was designed according to the acupuncture protocol used in Chinese clinical acupuncture practice. All acupuncture practitioners had undergone at least 8 years of acupuncture training and were qualified TCM doctors; indeed some were associate chief TCM doctors.

### Outcome measurements

Patients will be required to complete the Chinese version of the Nepean Dyspepsia Index (NDI), which has been translated according to the validated long version of the Nepean Dyspepsia Index (NDI) [25]. The reliability and validity of this index were tested in the first phase of the ***973 program***. Patients will also be asked to complete the Symptom Index of Dyspepsia and the Mos 36-item Short Form Health Survey (SF-36). The Symptom Index of Dyspepsia focuses on four symptoms, namely postprandial distension, early satiety, epigastric pain and heartburn. Each symptom is then graded as asymptomatic (0 point), mild (1 point), moderate (2 points) or severe (3 points). The SF-36 is a Chinese version of an English test [2] to assess the quality of life.

The main outcome measurements in this trial will include differences in the Nepean Dyspepsia Index (NDI) and the Symptom Index of Dyspepsia, as measured before randomization, 2 weeks and 4 weeks after randomization, and 1 month and 3 months after the completion of treatment. Secondary outcome measures will include differences in the SF-36 before randomization, 4 weeks after randomization, and 1 month and 3 months after completing treatment.

### Patient safety

All patients will undergo two routine tests of blood, urine and stool samples, as well as electrocardiogram (ECG) examination, blood biochemical tests (ALT, AST, BUN, Scr), and blood glucose (BGlu) tests before randomization and immediately after completing treatment. These tests will help identify and exclude patients with serious illnesses of the heart, liver and kidney or other severe disease. Furthermore, the results of these tests will allow the assessment of risks associated with acupuncture and itopride treatments.

Adverse events caused by acupuncture or itopride, as well as the remedies for these events, will be documented during the treatment and follow-up phases. Such adverse events may include bleeding, hematoma, fainting, serious pain and local infection.

### Quality control

All acupuncturists are required to undergo special training, including theoretical and practical lessons, before participating in the trial. All researchers and clinicians must understand the purpose and design of this trial, as well as treatment strategies and measures of quality. In addition, acupuncturists will be trained to use the central randomization method, complete the CRFs and electro-CRFs, locate the points and manipulate the needles. Acupuncturists who have completed the required training and have passed an examination will be considered qualified to assist with this trial. Several trained outcome assessors, who are blinded to group assignment, will be responsible for the outcome assessment. Additionally, to maintain accuracy and quality throughout our clinical study, audits will be conducted on CRF completion and compliance with standard operation procedures (SOPs). Finally, quality control and assurance will be presented in written form by trained monitors at every participating hospital once a month. Patient compliance and voluntary withdrawal from the study (including the reason for withdrawal) will be fully documented.

### Statistical analysis

Sample size was determined prospectively as described in a previous study that used similar outcome measurements [21]. The following formula was used to estimate sample size [26]:



A previous study reported [21] an improvement of 18.0 in the main outcome NDI index score after treatment with 50 mg itopride. In this study, we anticipate an improvement of no less than 15.0 after acupuncture treatment; however, there are no previous data regarding NDI scores for the treatment of FD using acupuncture. Accordingly, an improvement of 3.0 in the NDI scores of two groups will be considered minimally significant. Standard deviation was defined as 22.0, according to the previous study [21]. Calculations were performed using 90% power and a 5% significance level, resulting in an estimated 120 patients per group. Thus, we must enroll 720 patients in the six groups, to allow for a 15% withdrawal rate.

The two-sided null hypotheses to be tested include:

(1) The primary outcomes of the acupoint treatment groups will be equivalent to those of the itopride control group.

(2) The primary outcomes of the acupoint treatment groups will be equivalent to those of the non-acupoint control group.

(3) The primary outcomes of the acupoint treatment groups will be equivalent to each other.

We will test these hypotheses with a significance level of P < 0.05. The first step of the analysis will involve comparing the baseline characteristics of the groups. The second step will be to compare the efficacy of acupoint and itopride in the treatment of FD. The third step will involve comparing the acupoint treatment group with the non-acupoint control group. If acupoint treatment is more effective than non-acupoint treatment in the treatment of FD, the fourth step will involve comparing the efficacy of four acupoint treatments.

The analyses of baseline characteristics, as well as the primary, secondary and other outcomes, are based on the intention-to-treat (ITT) principle. Data distribution is expected to be normal owing to the large sample size. However, any skewed distribution data will be transformed prior to analysis. The repeated measures analysis will be used in the assessment of different time points (such as comparing NDI scores before randomization, 2 weeks and 4 weeks after randomization, and 1 month and 3 months after the completion of treatment). Missing values will be addressed using the method of last observation carried forward (LOCF) and multiple-imputation. We will perform sensitivity analysis to determine how these two methods affect the analytic results. General linear mixed models, adjusted for clinical site, will be used to study the effects of treatments on outcomes from baseline to 3 months. Additionally, time-to-event outcomes between groups will be compared using the log-rank test.

Analysis of data will be performed in a blinded manner by a designated statistician at the National Clinical Trial Center of Chinese Medicine (Chengdu GCP Center) in China. Statistical analyses will be performed using SPSS 15.0 statistics software (SPSS Inc, Chicago, Illinois, USA) and SAS 9.0 (SAS Institute Inc., Cary, North Carolina, USA).

## Discussion

The ***973 program***, also known as the National Basic Research Program of China (NBRPC), is the most important basic research program in China and the largest clinical trial investigating the specific physiological effects of acupoints.

The ***973 program ***consists of two clinical trials that attempt to clarify the specific effect of acupoints. One study focuses on the use of acupuncture to treat migraines [18], whereas the present study focuses on the use of acupuncture to treat FD.

The second part of the ***973 program ***will compare the efficacies of acupoint treatment, non-acupoint treatment and itopride treatment to determine if acupuncture is an effective treatment for FD. Furthermore, this study will compare different acupoint treatments to determine if specific acupoints and non-specific acupoints differ in efficacy. To our knowledge, this is the largest randomized, blinded placebo-controlled trial to examine the effects of acupuncture on FD, and one of the first trials to examine the traditional acupuncture theories of meridians and acupoints.

The theory of TCM and acupuncture strongly suggests that acupoints and non-acupoints differ in their abilities to promote specific physiological effects. Acupuncture is gradually becoming more accepted in countries beyond China, including in Western countries, as a potential treatment for a wide array of diseases. Indeed, this method has been used in clinical practice for many years. In recent years, differences in the "specific" and "non-specific" effects of acupoints have been examined by some Western studies [22,27,28]. According to TCM, there are differences between closely related meridians and other meridians, and there are also differences between specific acupoints (such as five transport points, alarm points, or transport points) and non-specific acupoints. Thus, a specific acupoint for the stomach meridian will be stimulated in group 1, a non-specific acupoint for the stomach meridian will be stimulated in group 2, alarm and transport points will be selected in group 3, and the gallbladder meridian (selected as being representative of other non-specific disease-related meridians, with a similar specific acupoint as that used in group 1) was stimulated in group 4. We have examined all relevant acupuncture literature and have analyzed the frequencies of acupoint during the first phase of our ***973 program***. The results indicate that the most commonly used acupoints are specific acupoints, such as Zhongwan (CV12, an alarm point), Zusanli (ST36, five transport points, in the meridian of the stomach), and Weishu (BL21, a transport point).

Most previous acupuncture trials were performed using semi-standardized acupuncture protocols. In this trial, we will restrict the number of acupoints and standardize the manipulation procedure. These actions have two merits: First, it is better to blind patients from the type of acupuncture they are receiving and second, some confounding factors (such as special rituals of acupuncture) will be excluded from the analysis, thereby making the acupuncture groups more comparable. However, this study design has its limitations; for example, the use of limited acupoints may not be an optimal acupuncture treatment protocol.

We choose itopride as the drug control because it has been shown to be an effective drug for FD. Furthermore, itopride caused fewer adverse events than did other gastrointestinal prokinetic agents [21]. Therefore, we will attempt to maintain a non-inferiority design because acupuncture is associated with no significant side effects. However, our trial will be limited by our inability to blind patients from knowing whether they are receiving acupuncture or itopride treatment. This factor may cause a high drop-out rate in the itopride group because patients will expect to receive acupuncture treatment when they join the trial. To prevent the expected high drop-out rate, patients in the itopride group will be provided with 10 sessions of free acupuncture after the entire trial has been completed.

Central randomization, a strict and complete form of randomization, will be used in this trial. This method not only provides rapid determination of group assignment, but also ensures adequate allocation concealment. In this trial, we will use transcutaneous electric acupoint stimulation (HANS) to avoid the performance bias generated by acupuncture practitioners. This method will provide accurate electric stimulation to ensure that every patient receives the same level of treatment.

We have devoted much effort to defining the non-acupoint control groups because the locations of non-acupoint treatments will differ from those described in the literature. Most Chinese studies have selected non-acupoints that lie beside the therapeutic acupoint, and halfway between the two lines or two acupoints [24]. However, reports in the Western literature have recorded the location of non-acupoints differently [29,30]. In our previous trial, we designed our two control groups according to the consensus of a panel of acupuncture experts. Thus, the non-acupoint control group in the current study was defined according to the first phase of our ***973 program***.

Briefly, this trial aims to determine if acupuncture is an effective treatment for FD. We will also assess differences in the specific effects of acupoints, as described in traditional theories. Certain procedures described herein, such as the randomization procedure, as controlled by central randomization system, and the standardized protocol for acupuncture manipulation, can be applied to future acupuncture studies.

## Competing interests

The authors declare that they have no competing interests.

## Authors' contributions

HZ, XPT, YL, FYL, SGY, XGL and YT contributed to the conception and design of the study. HZ and XPT drafted the manuscript. All authors contributed to the further writing of the manuscript. All authors read and approved the final manuscript.
